# Quantitative Evaluation of Iron-Containing Proteins Bound to Mesoporous Silica Microspheres by Inductively Coupled Plasma Mass Spectrometry and Confocal Laser Raman Microscopy

**DOI:** 10.3390/molecules30061252

**Published:** 2025-03-11

**Authors:** Shin-ichi Miyashita, Toshihiko Ogura, Shun-ichi Matsuura, Eriko Fukuda

**Affiliations:** 1 National Metrology Institute of Japan (NMIJ), National Institute of Advanced Industrial Science and Technology (AIST), 1-1-1 Umezono, Tsukuba 305-8563, Ibaraki, Japan; shinichi-miyashita@aist.go.jp; 2 Health and Medical Research Institute, National Institute of Advanced Industrial Science and Technology (AIST), 1-1-1 Higashi, Tsukuba 305-8566, Ibaraki, Japan; t-ogura@aist.go.jp; 3 Research Institute for Chemical Process Technology, National Institute of Advanced Industrial Science and Technology (AIST), 4-2-1 Nigatake, Miyagino-ku, Sendai 983-8551, Miyagi, Japan; matsuura-shunichi@aist.go.jp; 4 Cellular and Molecular Biotechnology Research Institute, National Institute of Advanced Industrial Science and Technology (AIST), 1-1-1 Higashi, Tsukuba 305-8565, Ibaraki, Japan

**Keywords:** iron-containing proteins, quantification, silica, microsphere, ICP-MS, confocal laser Raman microscopy

## Abstract

Inductively coupled plasma mass spectrometry (ICP-MS) is important in the biological and biochemical fields as it can quantify trace elements. Confocal laser Raman microscopy (CLRM), a powerful tool for the compositional analysis of biological samples, organic materials, and inorganic materials, can be used to analyze samples in aqueous solutions. Despite their analytical strength, the quantitative evaluation of proteins bound to mesoporous silica (SiO_2_) microspheres, which are promising candidates for drug delivery systems and vaccine carriers, has not been sufficiently explored. Therefore, we investigated the applicability of ICP-MS and CLRM to quantify lactoferrin (LF), a widely studied iron-containing protein bound to mesoporous SiO_2_ microspheres (SBA24). The bound LF amount was measured using ICP-MS, selectively monitoring iron derived from LF as a marker element, and CLRM. The results were compared with those obtained using a conventional bulk analysis technique. The amounts and trends of the signal intensities obtained using ICP-MS and CLRM agreed with each other and with the bulk analysis results. Our findings demonstrate that ICP-MS and CLRM are applicable for the quantitative evaluation of iron-containing proteins bound to SBA24. These methods offer a reliable platform for the quantification of biomolecules on microparticles and provide valuable insights for biomedical research and quality control in related industries.

## 1. Introduction

The design, synthesis, and testing of nano- and micron-sized particles are highly active research areas in materials science and, therefore, require the continuous development of analytical particle characterization techniques [1]. The primary particle properties include morphology, size, structure, composition, density, and porosity. Many studies have shown that among the various types of particles, porous particles have enhanced physicochemical properties and wide potential applications, including photocatalysis, drug delivery, photonic materials, batteries, absorbents, and fuel cells [2].

Porous silica (SiO_2_) particles find wide-scale applications as catalyst supports, adsorbents, molecular sieves, chemical sensors, and so on [3,4,5,6,7]. Some research (e.g., [8]) also found that mesoporous SiO_2_ (MPS) particles are very promising medical drug carriers. Owing to their unique properties, such as superior binding and adsorption, porous/mesoporous SiO_2_ particles have attracted considerable attention for various applications.

The binding/adsorption of nanoparticles (NPs) [9] or biomolecules, such as proteins [10], on porous microspheres has been extensively studied. For example, Kim et al. [9] produced mesoporous polymer microspheres with gold (Au) NPs inside their pores to observe the adsorption behavior of these NPs considering their surface functionality and porosity. Brunauer–Emmett–Teller (BET) experiments on Au/poly (ethylene glycol dimethacrylate-co-acrylonitrile) composite microspheres, used to measure the microsphere porosity, revealed that the adsorption of Au NPs into the pores kept the pore structure intact and made it more porous. Rafati et al. [10] used time-of-flight secondary ion mass spectrometry (ToF-SIMS) to investigate polymer microspheres for the controlled release of a therapeutic protein from an implantable scaffold. The ability of ToF-SIMS imaging to spatially image a polyvinyl alcohol (PVA) surfactant and protein adsorbed onto the surface of microspheres was demonstrated for the first time. Analytical techniques play a significant role in studying the properties of porous microspheres and in understanding the interactions between porous microspheres and NPs or biomolecules.

Inductively coupled plasma mass spectrometry (ICP-MS) is one of the most efficient, practical, and stable techniques of elemental analysis. ICP was used to ionize the atoms of the sample, which were then separated and identified based on their mass-to-charge ratios. It has been widely used for the analysis of metals and heteroatoms (e.g., P, S, Cl, Br, and I), particularly in the biological and biochemical fields, owing to its outstanding advantages of high sensitivity, low limit of detection, wide linear dynamic range, long-term stability, multi-element analysis capability, and high sample throughput [11,12]. For example, ICP-MS has been used to determine the target elemental composition of well-known iron (Fe)-containing proteins such as lactoferrin (LF) and transferrin [13,14,15,16]. However, its applicability to the element-based quantification of Fe-containing proteins bound to mesoporous silica (SiO_2_) microspheres is unclear.

Confocal laser Raman microscopy (CLRM) can analyze the composition of samples from the Raman scattering spectrum excited by irradiating a sample with laser light [17,18,19,20,21]. Furthermore, because it can analyze samples in aqueous solutions, it has been used in recent years to analyze cultured cells and organic materials [17,18,19,20,21]. Additionally, its capability to provide spatially resolved chemical imaging allows detailed analysis of heterogeneous samples. However, CLRM is inherently limited by the weak intensity of Raman scattering, typically 10^7^ to 10^9^ times smaller than Rayleigh scattering (elastic scattering), which can pose challenges for certain samples. Similar to ICP-MS, the potential of CLRM for the quantitative evaluation of Fe-containing proteins bound to mesoporous SiO_2_ microspheres remains underexplored.

In the present study, we investigated the applicability of ICP-MS and CLRM to the quantitative evaluation of LF bound to mesoporous SiO_2_ microspheres (SBA24) by measuring the amount of the bound LF using these techniques and comparing the results with those obtained by a bulk analysis technique used as a reference technique.

## 2. Results and Discussion

### 2.1. Element-Based Quantification of LF Bound to Mesoporous SiO_2_ Microspheres by ICP-MS

**Figure 1:**
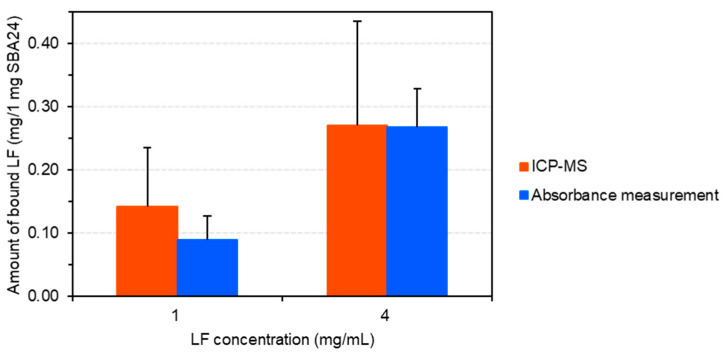
Quantification results of the protein (LF) bound to mesoporous SiO_2_ microspheres (1 mg SBA24) by element-based ICP-MS analysis and bulk analysis (absorbance measurement) techniques.

We quantitatively evaluated the effect of protein concentration on the adsorption of LF onto mesoporous SiO_2_ microspheres (SBA24) using ICP-MS based on a conventional calibration approach using an ion standard solution. The calibration curve for Fe standard solutions using four concentration points ranging from 0 µg/L to 100 µg/L was established with a correlation coefficient (R^2^) of 1.000 (Supplementary Figure S1). The regression equation was y = 4015x + 1361 for ^56^Fe. As shown in Figure 1, the amount of the bound LF (0.27 mg/1 mg SBA24) at the 4-mg/mL protein was notably higher than that (0.14 mg/1 mg SBA24) at the 1-mg/mL protein: the proportion of LF adsorbed on the pores of SBA24 at the 4-mg/mL protein was approximately 27 wt% (by weight of SBA24), which was 1.9 times higher than that adsorbed to SBA24 at the 1-mg/mL protein (ca. 14 wt%). Moreover, we calculated the amount of the bound LF per particle using the number of particles (8.2 × 10^7^ particles) in 1 mg SBA24, which was calculated using the mean particle mass (1.2 × 10^−8^ mg) measured by single-particle ICP-MS (spICP-MS) in our previous study [22]. As shown in Supplementary Figure S2, the resultant values were 1.7 pg/particle at the 1-mg/mL protein and 3.3 pg/particle at the 4-mg/mL protein.

To demonstrate the limitations of the present ICP-MS technique, we estimated the limit of detection (LOD) and the limit of quantification (LOQ) as the ratio of 3σ and 10σ value of the blank signal to each of the isotopic sensitivity, respectively. The resultant LOD and LOQ were 0.0019 mg/1 mg SBA24 (corresponding to 0.19 wt% by weight of SBA24) and 0.0064 mg/1 mg SBA24 (0.64 wt%), respectively. Furthermore, we calculated the amount of bound LF per particle using the number of particles (8.2 × 10^7^ particles) in 1 mg SBA24, as described above. The resulting LOD and LOQ values were 0.024 pg/particle and 0.079 pg/particle, respectively. There are various methods proposed for the quantification of LF in its soluble form [23]. Among them, an enzyme-linked immunosorbent assay (ELISA) is now considered as an ideal method for the detection of LF in solution. Some research groups proposed a sandwich ELISA method and reported the LOD values of 18 ng/mL [24] and 3.23 ng/mL [25] on a solution basis. However, the method is disadvantageous in that the price of the reagent kits is relatively expensive and the process of doing ELISA is laborious and time consuming [23]. In contrast, our proposed ICP-MS technique has the LOD value of 0.024 pg/particle on a single-particle basis, owing to the outstanding advantages of ICP-MS (high sensitivity, low LOD, etc.). Nonetheless, it is noted that the technique suffers from the disadvantage of potentially being influenced by multiple factors in the process of sample preparation and analysis, which can be clearly seen as the larger standard deviations of the mean values (i.e., larger error bars) as compared to those obtained from the absorbance measurement in Figure 1 and Supplementary Figure S2. It can be summarized that the present ICP-MS technique has LOD and LOQ for LF on the order of single micrograms per 1 mg SBA24 (i.e., tens of femtograms per particle), even though it can be influenced by multiple factors in the process of sample preparation and analysis.

### 2.2. Quantitative Evaluation of LF Bound to Mesoporous SiO_2_ Microspheres by Confocal Laser Raman Microscopy

**Figure 2:**
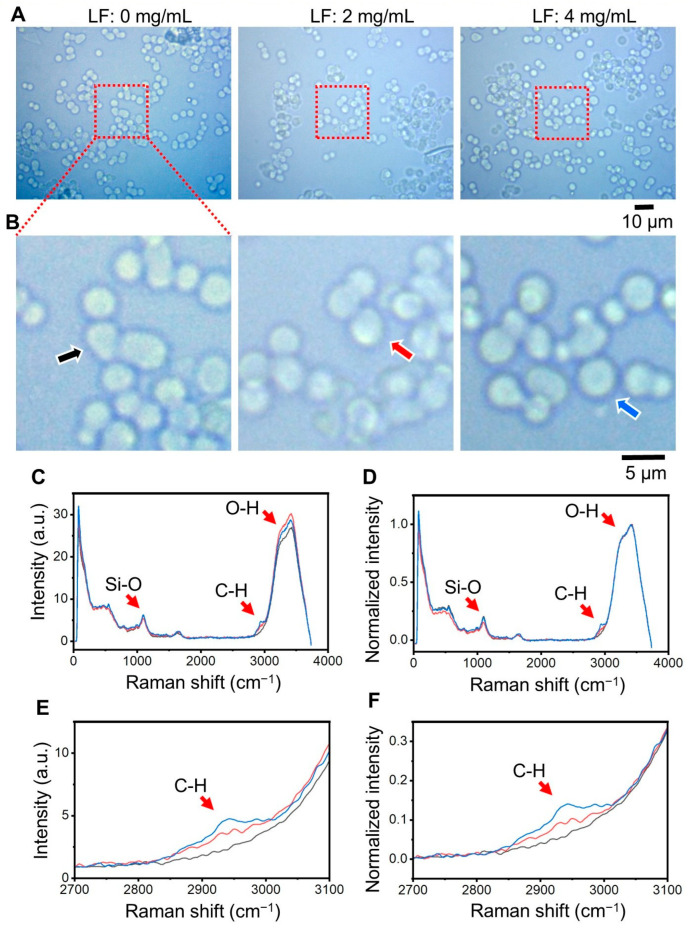
Optical microscope images and Raman spectra of the SBA24 particles with different LF concentrations. (**A**) Images of SBA24 particles in solution at LF concentrations of 0, 2, and 4 (mg/mL). (**B**) Enlarged images of the center of each (**A**) image. Arrows indicate SBA24 particles used for average Raman spectra. (**C**) Raman spectrum of SBA24 particles indicated by the arrow in each (**B**) image. Spectral peaks of Si–O bonds (1050 cm^−1^), O–H bonds of water (3100 cm^−1^ to 3800 cm^−1^), and C–H bonds of LF (2800 cm^−1^ to 3000 cm^−1^) can be seen. (**D**) Raman spectrum normalized using the average value of 2450–2550 cm^−1^ and the peak of O–H bonds of water. (**E**,**F**) Enlarged Raman spectrum and normalized Raman spectrum of the C–H bonds of LF area. LF concentrations of 0 mg/mL are indicated by a gray line, 2 mg/mL by a red line, and 4 mg/mL by a blue line. Scale bars, 10 µm in (**A**), 5 µm in (**B**).

The amount of LF bound to SBA24 was measured using CLRM. CLRM allows compositional analysis of samples by analyzing the Raman spectrum obtained when a sample is irradiated with laser light [17,18,19,20]. In particular, during the analysis of biological samples, the portions of lipids and proteins in the biological sample can be identified from peaks in the Raman spectrum. Here, we analyzed the Raman spectrum when LF, a type of protein, was adsorbed onto SBA24 (Figure 2). Figure 2A,B show optical microscopy images of SBA24 with LF added at concentrations of 0–4 mg/mL. SBA24 with diameters of 3 µm to 5 µm were dispersed in the aqueous solution. However, optical microscopy images showed no contrast and/or brightness intensity changes in SBA24 due to the LF concentration.

Next, we performed Raman spectral mapping of the observation range shown in Figure 2B and obtained the average Raman spectrum within SBA24 of Figure 2C, as indicated by the arrow in Figure 2B. To obtain the average Raman spectrum within the particle, the SBA24 area was masked (Supplementary Figure S3), and the average Raman spectrum within the mask was obtained. The Si–O bonds peak at 1050 cm^−1^ has almost the same intensity at each LF concentration. In this observation of SBA24, SBA24 in solution was enclosed between a slide glass and a cover glass. In the Raman spectra of the cover glass, slide glass, and SBA24 alone, there is a signal of Si–O bonds at 900–1200 cm^−1^, but the peak position and shape are different (Supplementary Figure S4). Therefore, it is expected that the signal of Si–O bond of SBA24 is mixed with the signals of the cover glass and slide glass. On the other hand, differences due to concentration were observed in the protein Raman peak at 2800 cm^−1^ to 3000 cm^−1^ [21]. This region is attributed to hydrogen stretching vibrations of the methyl and methylene (v(C–H)) groups. The peaks with maxima at 2939–2950 cm^−1^ for these groups are not as typical for proteins as, for example, the amide peak. However, it is an excellent marker for the presence of biological material including proteins. In addition, the spectrum of O–H bonds group in water from 3100 cm^−1^ to 3800 cm^−1^ showed intensity variations that were independent of LF concentration. This is likely due to variability in each observation, as this is the average spectrum for the entire SBA24 particle. If normalization is performed using the average value of 2450–2550 cm^−1^, where the Raman intensity is at its lowest, and the peak of O–H bonds of water, the peak shape of the O–H signal will completely overlap (Figure 2D). Figure 2E,F shows a plot of the Raman spectrum and Normalized Raman spectrum of the C–H signal of LF enlarged from 2700 cm^−1^ to 3100 cm^−1^. When the LF concentration was 0 mg/mL, no peaks were observed (Figure 2D,F, gray line). In contrast, as the LF concentration increased from 2 mg/mL to 4 mg/mL, the peak around 2950 cm^−1^ increased (Figure 2D,F, red and blue lines). In the normalized Raman spectrum, the baseline is aligned, and the LF-derived C–H bonds peak is clear (Figure 2F).

**Figure 3:**
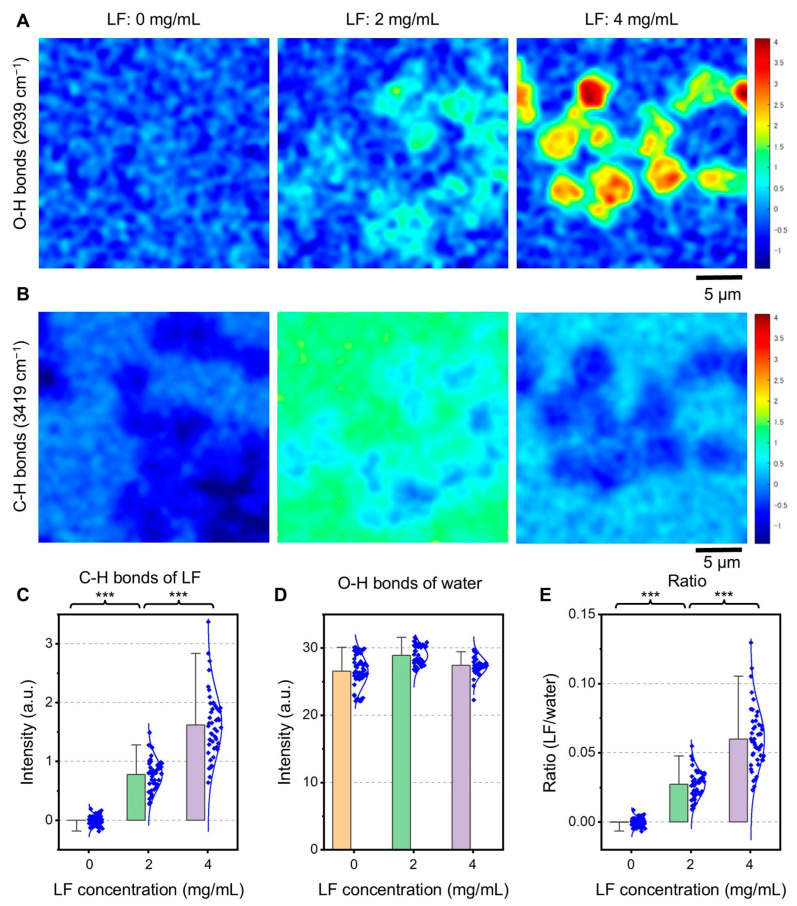
Raman spectrum map of SBA24 particles at each LF concentration. (**A**) Raman map of the C–H bonds peak (2939 cm^−1^) at LF concentrations of 0, 2, and 4 (mg/mL). (**B**) Raman map of the peak of O–H bonds from water (3419 cm^−1^) at LF concentrations of 0, 2, and 4 (mg/mL). (**C**) Bar graph of the C–H bonds peak (2939 cm^−1^) at each LF concentration. Blue dots indicate all data used in the calculation. (**D**) Bar graph of the O–H bonds Raman peak at each LF concentration. (**E**) Bar graph of the ratio of Raman peak values of C–H and O–H bonds. Signals marked by stars are significantly different by *t*-test: *** (*p* < 0.001). Scale bars, 5 µm in (**A**,**B**).

Figure 3A shows the Raman spectrum map of the C–H bonds peak at 2939 cm^−1^. When the LF concentration was 0 mg/mL, no high-intensity areas were observed, and the image was almost noisy (Figure 3A, left side). When the LF concentration was 2 mg/mL, a small peak corresponding to LF incorporated into SBA24 was observed (Figure 3A, center). Furthermore, when the LF concentration was increased to 4 mg/mL, a region of strong C–H bonds peak was observed within SBA24 (Figure 3A, right panel), indicating accumulation of LF within SBA24. Figure 3B shows a Raman spectrum map of the water peak at 3419 cm^−1^. In this map, the water peak of SBA24 was slightly reduced and appeared dark blue. This was due to the slightly reduced amount of water present within SBA24 compared to the surrounding water.

From the Raman spectrum map images of each LF concentration, 47–55 SBA24 particles were selected, and the Raman intensities of C–H bonds (LF) and O–H bonds (water) for each particle were obtained (Figure 3C,D). When the LF concentration was 0 mg/mL, the protein intensity was almost 0, but as the LF was increased to 2 mg/mL and 4 mg/mL, the Raman intensity also increased from 0.78 to 1.6 (Figure 3C). The t-test shows that the significant difference in C–H bonds peak between each LF concentration is very high at *p* < 0.001. In contrast, the Raman peak intensity of water did not differ with concentration (Figure 3D). There was some variation in the spectral intensity, which was attributed to the variation in the water content of SBA24. To correct for this variation in the water content of SBA24, the ratio of the protein peak to the water peak was measured, and the LF concentration was compared (Figure 3E). This result was almost the same as that of the protein peak intensity, confirming that the effect of variation due to water content in SBA24 was small.

### 2.3. Quantification of LF Bound to Mesoporous SiO_2_ Microspheres by a Bulk Analysis Technique

The effect of protein concentration on the adsorption of LF onto SBA24 was quantitatively evaluated using a bulk analysis technique (used as a reference) (Figure 1). The amount of the bound LF (0.27 mg/1 mg SBA24) at the 4-mg/mL protein was notably higher than that (0.09 mg/1 mg SBA 24) at the 1-mg/mL protein: the proportion of LF adsorbed on the pores of SBA24 at the 4-mg/mL protein was approximately 27 wt% (by weight of SBA24), which was 3.0 times higher than that adsorbed to SBA24 at the 1-mg/mL protein (ca. 9 wt%).

### 2.4. Comparison of the Quantification Results Obtained from the Above-Mentioned Techniques

The amount of bound LF was measured using both ICP-MS and CLRM, and the results were compared with those obtained using bulk analysis (used as a reference technique). As shown in Figure 1, the amounts of bound LF obtained using ICP-MS and bulk analysis were within the analytical uncertainty. At the 4-mg/mL protein, for example, the amounts of the bound LF were (0.27 ± 0.16) mg/1 mg SBA24 and (0.27 ± 0.06) mg/1 mg SBA24, respectively, as the mean of three subsamples (*n* = 3) ± the corresponding standard deviation. Furthermore, the signal intensities obtained from the bound LF using CLRM (Figure 3) agreed well with those obtained using ICP-MS and bulk analysis (Figure 1). Thus, ICP-MS and CLRM are applicable to the quantitative evaluation of Fe-containing proteins bound to SBA24. These techniques have considerable potential for the quantitative evaluation of diverse biological objects, such as metalloproteins and lipids [26], bound to SBA24 in various application fields.

As shown in Figure 1, the amount of bound LF at 4-mg/mL protein was significantly higher than that at the 1-mg/mL protein. This suggests that the amount of protein binding depends considerably on LF concentration. Thus, LF-bound SBA24 can be easily prepared by successful binding of LF molecules to the pores. LF is positively charged under buffer conditions (pH 7.4) because its theoretical isoelectric point is 8.67; therefore, it is thought that LF adsorption to the negatively charged silica surface is facilitated by electrostatic interactions.

## 3. Materials and Methods

### 3.1. Materials

Mesoporous SiO_2_ microspheres (SBA24 with a pore diameter of 23.5–23.6 nm) were synthesized based on previously reported methods [27,28]. The dried SBA24 powder was stored at 20–25 °C in a sealed desiccator until further use. Dried LF powder (product code 123-04124) was purchased from FUJIFILM Wako Pure Chemical Corporation (Osaka, Japan). The 10× Tris-buffered saline (TBS) buffer (pH 7.4; product code 317-90175) from Nippon Gene Corporation (Toyama, Japan) was diluted 10-fold with ultrapure water to prepare TBS. The solution was then used to suspend SBA 24 and dissolve LF.

For creating a four-point calibration curve in ICP-MS, ion standard solutions of Fe with different concentrations of 0–100 µg/L in 2% HNO_3_ and that of yttrium (Y) with a stock concentration of 125 µg/L in 2% HNO_3_ were gravimetrically prepared from 1000 mg/L single-element standard solutions (Kanto Chemical Corporation, Tokyo, Japan). Nitric acid (HNO_3_) and hydrochloric acid (HCl) (ultrapure grade; Kanto Chemical Corporation, Tokyo, Japan) were used for the acid digestion of LF bound to SBA24 and to prepare solutions such as 2% HNO_3_.

### 3.2. Sample Preparation

Approximately 1 mg of dried SBA24 powder was weighed in a tube. One milliliter of aqueous TBS buffer (pH 7.4) was added to the tube and rigorously vortexed twice for a few seconds each. The resultant suspension was gently rotated at 20–25 °C for 5 min. The SBA24 suspension was centrifuged at 12,000–19,000× *g* for 1 min at 20 °C, and the remaining pellet was used as TBS-equilibrated SBA24 for further experiments.

LF was used as a representative Fe-containing protein for binding to SBA24. A bottle of LF stored in a refrigerator was left to stand for 15–30 min before being returned to 20–25 °C. Appropriate amounts of LF (1 and 4 mg) were added to each tube. One milliliter of TBS was added to each tube, gently vortexed for 3 s, and slowly rotated for 30 min at 20–25 °C for complete dissolution. Thereafter, the resultant solution was centrifuged at 19,000× *g* for 5 min at 20 °C. The supernatant (i.e., the dissolved protein fraction) was transferred to a new tube and used as the protein solution to prepare LF-bound SBA24.

The adsorption of LF onto the pores of SBA24 was performed by combining 1 mL of a protein solution containing LF (0, 1, and 4 mg) with 1 mg of TBS-equilibrated SBA24. The LF-SBA24 mixtures were gently agitated using a rotator for 10 min at 20 °C and centrifuged at 20,000× *g* for 1 min at 20 °C. After centrifugation, LF-bound SBA24 was rinsed twice with 1 mL of TBS for the subsequent quantitative evaluation of LF bound to SBA24 by ICP-MS and CLRM.

### 3.3. ICP-MS Quantification Method of LF Bound to Mesoporous SiO_2_ Microspheres

Element-based quantification of LF bound to SBA24 was performed by ICP-MS using Fe as a marker of LF. LF bound to SBA24 was subjected to acid digestion according to a previously reported method [29]. Briefly, LF-bound SBA24 was dispersed in TBS (1 mL). Three subsamples (approximately 0.3 g each) taken from each dispersion were accurately weighed into different tubes, and 2 mL of aqua regia (a mixture of HNO_3_ and HCl in a 1:3 ratio) was added to each tube. The tube was shaken vigorously and sonicated in a Branson Ultrasonic Bath (M5800H-J; Branson Ultrasonics Corporation, Brookfield, CT, USA) for 10 min at 20–25 °C to obtain a homogeneous dispersion. Thereafter, the sample was diluted to 10 mL with 2% HNO_3_. The amount of Fe in the sample was determined using a quadrupole ICP-MS instrument (Agilent 7700x ICP-MS; Agilent Technologies, Santa Clara, CA, USA) equipped with an ICP torch with an injector tube diameter of 2.5 mm, a conventional MicroMist nebulizer, and a Scott double-pass spray chamber cooled at 2 °C in combination with the Agilent Integrated Sample Introduction System (ISIS). The ICP-MS instrument was tuned daily using a tuning solution containing 1 µg/L each of Li, Co, Y, Ce, and Tl in 2% HNO_3_ for optimum signal intensity and stability. The typical operating conditions of the ICP-MS instrument are listed in Table 1.

To determine the amount of Fe in the LF-bound SBA24 by ICP-MS, external calibration with internal standard correction was applied to compensate for physical interference, correct for variations in the instrument response as the analysis proceeded (signal drift), and calculate the analyte (Fe) concentrations of the samples [30]. For this purpose, Y with a monoisotopic mass of 89 was chosen as the internal standard element because it is usually not present at significant levels. The stock solution of Y (125 µg/L in 2% HNO_3_) was added to each sample and calibration standard solution at the final concentration of approximately 1.2 µg/L. A calibration curve of Fe was composed of four concentration points (0 µg/L, 1 µg/L, 10 µg/L, and 100 µg/L in 2% HNO_3_). The amount of Fe determined by ICP-MS with external calibration and internal standard correction was converted into the amount of LF using the percentage of Fe (0.029%) as reported by the manufacturer.

### 3.4. Measurement Condition of LF Bound to Mesoporous SiO_2_ Microspheres by Confocal Laser Raman Microscopy

The SBA24 suspension of 5 μL was dropped onto a slide glass, and the top was sealed with a cover glass by sandwiching 5 µm-thick double-sided tape. The samples were observed under CLRM using a 532 nm Nd:YAG laser (alpha300R; WITec, Ulm, Germany). The spectra were acquired using a Peltier-cooled charge-coupled device detector (DV401-BV, Andor, UK) at 600 gratings/mm (UHTS 600; WITec). Raman data were analyzed using the WITec suite (version 5.0; Lab Co., Northampton, MA, USA) and MATLAB R2023a (Math Works Incorporated, Natick, MA, USA). For observation of MNT-1 cells, a 25 µm × 25 µm area at the cell center was scanned at 100 pixels × 100 pixels using a 50 × objective lens at laser intensities of 20 mW at 50 ms for each pixel. This device uses an optical fiber with a diameter of 100 μm, and the core diameter of approximately 20 μm acts as a pinhole. From the Raman map images of SBA24 with LF concentrations between 0 mg/mL and 4 mg/mL, 55 particles (0 mg/mL), 50 particles (2 mg/mL), and 47 particles (4 mg/mL) were picked, and the outline of each particle was manually masked. The average Raman spectrum within a particle was calculated by averaging the Raman spectra of each pixel contained within the mask and dividing it by the number of pixels in the mask. The t-test for the peak values of C–H bonds and the ratio to O–H bonds between each LF concentration was calculated using Origin Pro 2024b (OriginLab Corporation, Northampton, MA, USA).

### 3.5. Quantification of LF Bound to Mesoporous SiO_2_ Microspheres by a Bulk Analysis Technique

To quantify LF bound to SBA24 by bulk analysis, the adsorption of LF on the pores of SBA24 was performed by combining 1 mL of a protein solution containing LF (1 mg and 4 mg) with 1 mg of TBS-equilibrated SBA24. The LF-SBA24 mixtures were gently agitated using a rotator for 10 min at 20 °C and centrifuged for 1 min at 20,000× *g*, and then the supernatant was recovered. To determine the amount of LF bound to SBA24, the protein concentration in the first supernatant was spectrophotometrically determined using a Pierce 660 nm Protein Assay (Thermo Fisher Scientific, Rockford, IL, USA). This assay utilizes a dye–metal complex that interacts with specific amino acid residues under acidic conditions, resulting in a detectable color change at 660 nm, which correlates with the protein concentration. The LF concentration was evaluated by measuring protein absorbance at 660 nm using an absorbance microplate reader (SpectraMax iD3; Molecular Devices, LLC., San Jose, CA, USA). The amount of LF adsorbed onto SBA24 was determined by subtracting the concentration of LF remaining in the supernatant from that before binding.

## 4. Conclusions

According to the results obtained in this study, ICP-MS and CLRM are applicable to the quantitative evaluation of Fe-containing proteins bound to mesoporous SiO_2_ microspheres. They have considerable potential for the quantitative evaluation of diverse biological objects, such as proteins and lipids [26], bound to mesoporous SiO_2_ microspheres in various fields, including drug delivery systems, sustained-release drugs administered in vivo, vaccine carriers, and biosensors.

The ICP-MS analysis revealed that the protein bound to SBA24 was 1.7–3.3 pg/particle, and CLRM successfully detected and quantitatively evaluated this protein. Colorimetric methods, such as bicinchoninic acid (BCA) protein assays, are simple to operate and widely used but have limited specificity, sensitivity, background noise resistance, and structural information. Although CLRM and ICP-MS generally offer superior performance, this study shows their applicability in quantifying proteins bound to microparticles, addressing their limitations, and providing complementary advantages for precise protein analysis.

In a previous study [22], we investigated the applicability of spICP-MS for the measurement of micrometer-sized SiO_2_ microspheres and the detection of Fe-containing proteins (i.e., LF and transferrin) bound to mesoporous SiO_2_ microspheres. Based on the results obtained, we conclude that spICP-MS is applicable to the particle size measurement of non-porous/mesoporous SiO_2_ microspheres and has considerable potential for element-based detection and qualification of proteins bound to mesoporous SiO_2_ microspheres in a variety of applications. In the near future, we will continue to investigate the potential of element-based quantification of proteins bound to mesoporous SiO_2_ microspheres.

## Figures and Tables

**Table 1:** Typical operating conditions of the ICP-MS instrument.

Parameter	Setting
Plasma and sampling conditions	
RF power	1550 W
Plasma gas flow rate	15 L/min
Auxiliary gas flow rate	0.90 L/min
Carrier (nebulizer) gas flow rate	1.08 L/min
Nebulizer pump	0.10 rps
Sampling position	10.0 mm
Cell gas (He) flow rate	5.0 mL/min
Data acquisition	
Scanning mode	Peak hopping
Data points	3 points/peak
Dwell time	0.6 s/point
Repetition	10 times
Monitored isotope	^56^Fe, ^89^Y

## Data Availability

Data presented in this study are available upon request from the corresponding author. The data is not publicly available due to privacy concerns.

## Supplementary Materials

The following supporting information can be downloaded at: https://www.mdpi.com/article/10.3390/molecules30061252/s1, Figure S1: Calibration curve for Fe standard solutions using four concentration points ranging from 0 µg/L to 100 µg/L, having a correlation coefficient (R^2^) of 1.000 with an equation of y = 4015x + 1361. cps = counts per second; Figure S2: Quantification results of the protein (LF) (in terms of amounts per particle) bound to mesoporous SiO_2_ microspheres by element-based ICP-MS analysis and bulk analysis (absorbance measurement) techniques using the number of particles (8.2 × 10^7^ particles) in 1 mg SBA24 (calculated with the mean particle mass of 1.2 × 10^−8^ mg determined by spICP-MS); Figure S3: Mask images for calculating the Raman spectrum of SBA24 particles. Images of each mask were used to calculate the Raman spectrum at LF concentrations of 0, 2, and 4 (mg/mL). The average value of the Raman spectrum for each pixel in the mask was calculated to obtain the result shown in Figure 2C; Figure S4: Raman spectrum of the slide glass, cover glass, and SBA24 alone. A signal of Si–O bonds is seen at 900–1200 cm^−1^, but the peak position and shape are different for each. The laser power for the measurement was 5 mW for the glass slide and cover glass, and 20 mW for SBA24. The measurement time was 1 s, and 50 measurements were taken and averaged.

## Abbreviations

The following abbreviations are used in this manuscript:

ICP-MS	Inductively coupled plasma mass spectrometry
CLRM	Confocal laser Raman microscopy
NPs	Nanoparticles
BET	Brunauer–Emmett–Teller
ToF-SIMS	Time-of-flight secondary ion mass spectrometry
PVA	Polyvinyl alcohol
spICP-MS	Single-particle ICP-MS
LOD	Limit of detection
LOQ	Limit of quantification
