# Supplementary material for: Quantitative Evaluation of Iron-Containing Proteins Bound to Mesoporous Silica Microspheres by Inductively Coupled Plasma Mass Spectrometry and Confocal Laser Raman Microscopy
Source: Molecules. 2025 Mar 11;30(6):1252. doi: 10.3390/molecules30061252 (PMC11944308; doi:10.3390/molecules30061252)
Supplement: Supplementary file 1 [file molecules-30-01252-s001.zip › Supplementary_Figure_S1.docx]

| 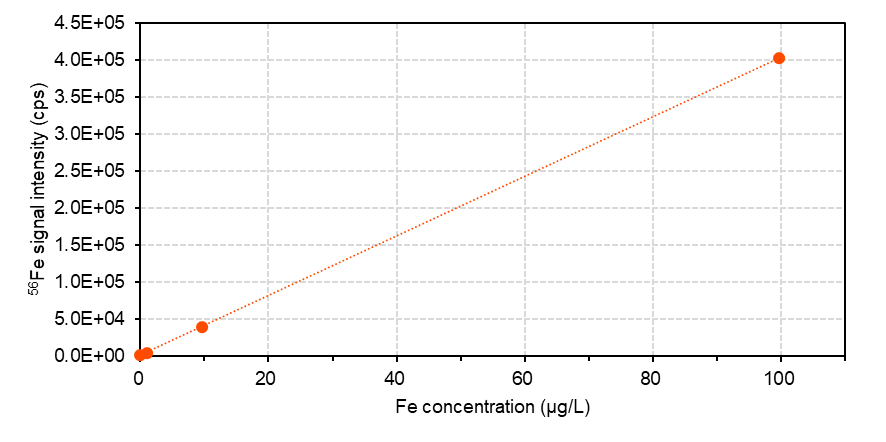 |
| --- |
| **Figure S1**. Calibration curve for Fe standard solutions using four concentration points ranging from 0 µg/L to 100 µg/L, having a correlation coefficient (R^2^) of 1.000 with an equation of y = 4015x + 1361. cps = counts per second. |
