# Supplementary material for: Quantitative Evaluation of Iron-Containing Proteins Bound to Mesoporous Silica Microspheres by Inductively Coupled Plasma Mass Spectrometry and Confocal Laser Raman Microscopy
Source: Molecules. 2025 Mar 11;30(6):1252. doi: 10.3390/molecules30061252 (PMC11944308; doi:10.3390/molecules30061252)
Supplement: Supplementary file 1 [file molecules-30-01252-s001.zip › Supplementary_Figure_S2.docx]

| 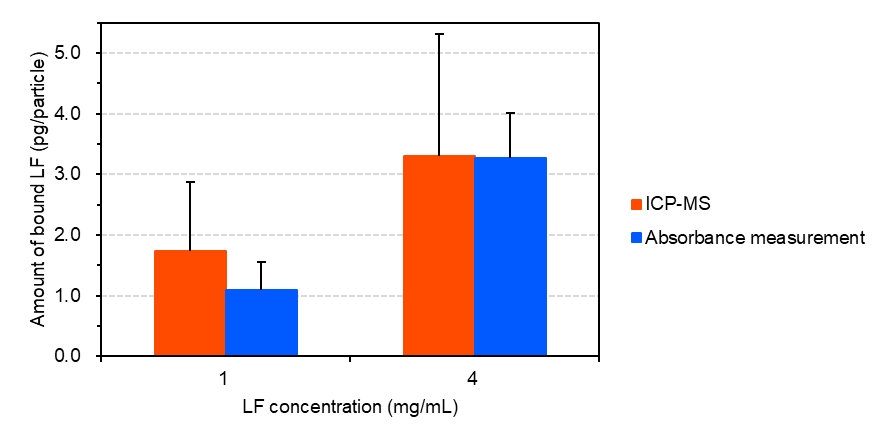 |
| --- |
| **Figure S2**. Quantification results of the LF (in terms of amounts per particle) bound to mesoporous SiO_2_ microspheres by element-based ICP-MS analysis and bulk analysis techniques using the number of particles (8.2 × 10^7^ particles) in 1 mg SBA24 (calculated with the mean particle mass of 1.2 × 10^−8^ mg determined by single-particle ICP-MS). |
