# Supplementary material for: Quantitative Evaluation of Iron-Containing Proteins Bound to Mesoporous Silica Microspheres by Inductively Coupled Plasma Mass Spectrometry and Confocal Laser Raman Microscopy
Source: Molecules. 2025 Mar 11;30(6):1252. doi: 10.3390/molecules30061252 (PMC11944308; doi:10.3390/molecules30061252)
Supplement: Supplementary file 1 [file molecules-30-01252-s001.zip › Supplementary_Figure_S3.docx]

| 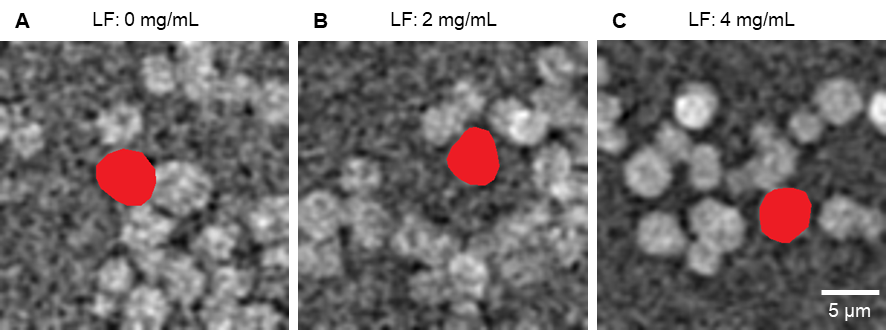 |
| --- |
| **Figure S3**. Mask images for calculating the Raman spectrum of SBA24 particles. Images of each mask used to calculate the Raman spectrum at LF concentrations of 0, 2, and 4 (mg/mL). The average value of the Raman spectrum for each pixel in the mask was calculated to obtain the result shown in Figure 2(C). |
