# Supplementary material for: Quantitative Evaluation of Iron-Containing Proteins Bound to Mesoporous Silica Microspheres by Inductively Coupled Plasma Mass Spectrometry and Confocal Laser Raman Microscopy
Source: Molecules. 2025 Mar 11;30(6):1252. doi: 10.3390/molecules30061252 (PMC11944308; doi:10.3390/molecules30061252)
Supplement: Supplementary file 1 [file molecules-30-01252-s001.zip › Supplementary_Figure_S4.docx]

| 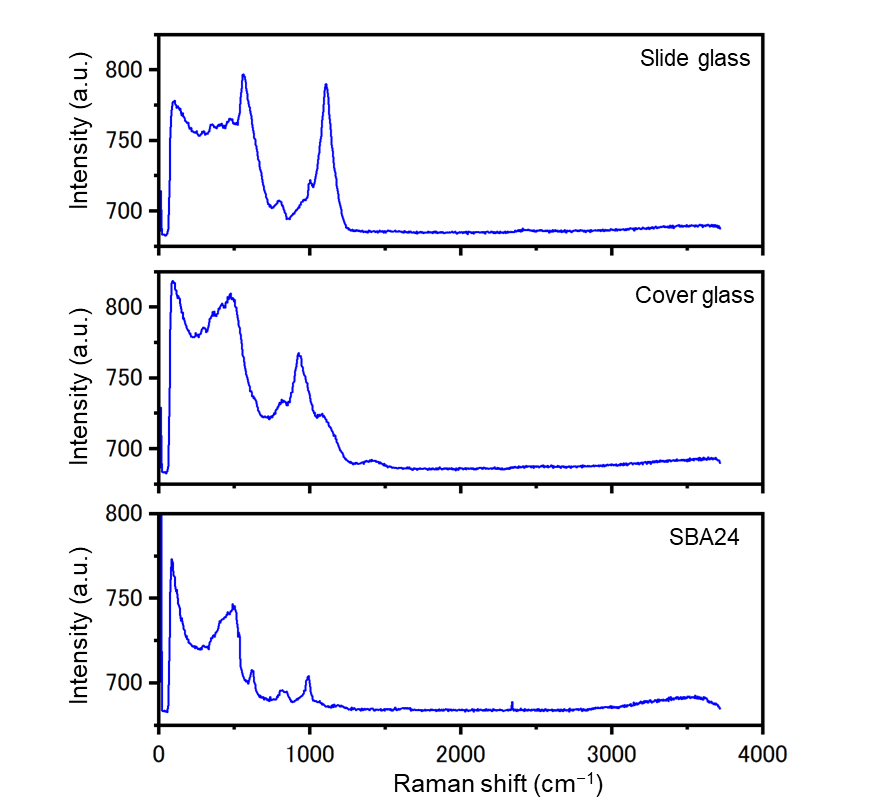 |
| --- |
| **Figure S4**. Raman spectrum of the slide glass, cover glass, and SBA24 alone. A signal of Si-O bonds is seen at 900–1200 cm^−1^, but the peak position and shape are different for each. The laser power for the measurement was 5 mW for the glass slide and cover glass, and 20 mW for SBA24. The measurement time was 1 second, and 50 measurements were taken and averaged. |
